# Lipoprotein apheresis affects the concentration of extracellular vesicles in patients with elevated lipoprotein (a)

**DOI:** 10.1038/s41598-024-51782-5

**Published:** 2024-02-02

**Authors:** Joanna Marlęga-Linert, Aleksandra Gąsecka, Edwin van der Pol, Agnieszka Kuchta, Krzysztof J. Filipiak, Marcin Fijałkowski, Marcin Gruchała, Rienk Nieuwland, Agnieszka Mickiewicz

**Affiliations:** 1 First Chair and Department of Cardiology, Medical University of Gdansk, Gdańsk, Poland; 2 1St Chair and Department of Cardiology, Medical University of Warsaw, Warsaw, Poland; 3 Amsterdam Vesicle Center and Laboratory of Experimental Clinical Chemistry, Amsterdam University Medical Centres, Amsterdam, The Netherlands; 4 Biomedical Engineering and Physics, Amsterdam University Medical Centres, Amsterdam, The Netherlands; 5 Department of Clinical Chemistry, Medical University of Gdansk, Gdańsk, Poland; 6 Institute of Clinical Sciences, Maria Skłodowska-Curie Medical Academy in Warsaw, Warsaw, Poland; 7 Department of Hypertensiology, Angiology and Internal Medicine, Poznan University of Medical Sciences, Poznań, Poland

**Keywords:** Biochemistry, Biophysics, Cardiology, Diseases, Medical research

## Abstract

Lipoprotein apheresis (LA) is a therapeutic option for hyperlipoproteinemia(a) (hyper-Lp(a)) and atherosclerotic cardiovascular disease (ASCVD). LA improves blood rheology, reduces oxidative stress parameters and improves endothelial function. The underlying molecular mechanisms of LA beneficial effects are unknown, but it has been suggested that LA exhibits multiple activities beyond simply removing lipoproteins. We hypothesized that LA removes not only lipoproteins, but also extracellular vesicles (EVs). To test this hypothesis, we performed a prospective study in 22 patients undergoing LA for hyper-Lp(a) and ASCVD. Different EVs subtypes were measured before and directly after LA, and after 7 days. We used calibrated flow cytometry to detect total particle concentration (diameter >  ~ 100 nm), total lipoproteins concentration (diameter > 200 nm, RI > 1.51), total EV concentration (diameter > 200 nm, RI < 1.41), concentrations of EVs derived from erythrocytes (CD235a+; diameter > 200 nm, RI < 1.41), leukocytes (CD45+; diameter > 200 nm, RI < 1.41) and platelets (CD61+, PEVs; diameter > 200 nm, RI < 1.41). LA reduced the concentrations of all investigated EVs subtypes and lipoproteins. Lp(a) concentration was lowered by 64.5% [(58% – 71%); p < 0.001]. Plasma concentrations of EVs > 200 nm in diameter derived from platelets (CD61 +), leukocytes (CD45+) and erythrocytes (CD235a+) decreased after single LA procedure by 42.7% [(12.8–54.7); *p* = 0.005], 42.6% [(29.7–54.1); *p* = 0.030] and 26.7% [(1.0–62.7); *p* = 0.018], respectively, compared to baseline. All EV subtypes returned to the baseline concentrations in blood plasma after 7 days. To conclude, LA removes not only Lp(a), but also cell-derived EVs, which may contribute to LA beneficial effects.

## Introduction

Lipoprotein (a) (Lp(a)) is an LDL-like particle, which concentration is independently and linearly associated with increased risk of myocardial infarction, stroke, cardiovascular (CV) death and aortic stenosis progression^1–11^. Currently, there are no clinically introduced, commercially available and approved medications that can efficiently lower Lp(a) blood concentration. Although proprotein convertase subtilisin/kexin 9 (PCSK9) inhibitors can reduce Lp(a) concentrations along with cardiovascular risk, they are not registered to treat isolated hyper-Lp(a)^7^. New agents including pelacarsen, olpasiran, SLN360 and muvalaplin which target apo(a) are currently being investigated in clinical trials.

Alternatively, Lp(a) blood concentration of can be reduced by lipoprotein apheresis (LA), which is currently used to treat patients with elevated Lp(a) concentrations and progressive atherosclerotic cardiovascular disease (ASCVD)^12–20^. LA decreases Lp(a) concentration by 73% and reduces the rate of major adverse CV events (MACE) by 86%^21,22^. The lipid-lowering effect has been suggested to be the main mechanism responsible for this clinical benefits of LA. However, LA exhibits also pleiotropic effects, which act in concert with its lipid-lowering mode of action^23–25^. LA improves blood rheological properties by decreasing its viscosity^26^, reduces oxidative stress and microalbuminuria, increases flow-mediated vasodilation, and improves endothelial function by affecting circulating endothelial cells and progenitor cells^27^. LA also mobilizes adult stem cells and lowers the expression of stress response genes. Additionally, LA reduces the expression of microRNAs involved in atherosclerosis development and progression^28–30^. However, there is lack of data about the effect of LA on the concentrations of circulating extracellular vesicles (EVs). EVs are cell-derived, membrane-enclosed particles that have heterogeneous properties and can be divided into subgroups based on their size, origin, or formation process^31^. EVs have emerged as mediators of intercellular communication by transporting various molecules, including proteins, lipids, nucleic acids, cytokines, chemokines, and signaling ligands between cells. EVs also contribute to homeostasis maintenance and are involved in the pathophysiology of inflammation, thrombosis, and neo-angiogenesis, all of which underly ASCVD. Monitoring changes in plasma EV concentrations can be used as a tool to assess the severity and progression of ASCVD^32–34^. Therefore, EVs may serve as novel CV biomarkers^35–38^. For example, concentration of platelet-derived EVs was higher in patients with acute coronary syndrome than those in the control group^39,40^. Increased concentrations of EVs were also found in patients with familial hypercholesterolemia (FH) compared to non-FH patients^41^. LA therapy has been shown to reduce EV concentration in patients with FH^42^. Recently, our understanding of EVs role has evolved from signaling markers to important effectors of intracellular communication. Therefore, EVs are increasingly being investigated as new pharmacological targets, giving promise for a new direction of targeted therapy.

To our knowledge, we are the first to studywhether LA can affect concentrations of various EV subtypes in patients with hyper-Lp(a) and ASCVD. We hypothesized that LA procedure may not only remove lipoproteins but also remove EVs, and their removal may be associated with reducing the risk of CVD.

## Materials and methods

This prospective study was conducted at the First Department of Cardiology, Medical University of Gdansk, Poland. The study protocol was designed in compliance with the Declaration of Helsinki and approved by the Ethics Committee of the Medical University of Gdansk (Approval Number KB/428/2018-2022). All participants provided written, informed consent.

We included 22 patients (8 females and 14 males) who underwent biweekly LA procedures in 2022. LA therapy was initiated based on the following recommendations: hyper-Lp(a) with an Lp(a) concentration > 100 mg/dl in patients with documented ASCVD.

All patients received the standard treatment according to the guidelines, including the maximal tolerated dose of statins in combination with ezetimibe. None of them was treated with PCSK9 inhibitors. The data collected included demographics (age, sex), body mass index, Lp(a) concentration at diagnosis, and cardiovascular risk factors (including familial hypercholesterolemia, chronic kidney disease, smoking, diabetes, and arterial hypertension). Clinical and biochemical characteristics of the study group is presented in Supplemental File 1. The patient diagnosed with chronic kidney disease had GFR of 53 ml/min/1.73 m2. Biochemical parameters at baseline and immediately before and after the LA procedure, were performed at the local laboratory and included lipid, biochemical, and coagulation profiles.

### Lipoprotein apheresis procedures

Patients underwent regular LA, with cascade filtration technique (MONET/Fresenius) which is the method of selective, extracorporeal removal of pro-atherosclerotic lipid particles. In the first step, the MONET system separates plasma from blood cells. In the second step, plasma is transferred through a specific membrane capillary filter, with high permeability for proteins < 100 kDa (90%) and low permeability for proteins > 1000 kDa. The manufacturer does not provide details on pore size of the filter, as the membranes are characterized via their sieving coefficients. In practice, the MONET system allows albumin, HDL, and smaller immunoglobulins to pass, whereas Lp(a), LDL, VLDL and chylomicrons, are retained and thereby removed from the plasma^43^.

All procedures were performed via peripheral venous access. Acid citrate dextrose and heparin were used as the anticoagulants.

### EV blood collection and handling

Peripheral venous blood samples were collected from fasting patients according to recent guidelines to study EVs^44^. Each patient had blood collected at three points in time: immediately before and after a single LA procedure, and then 7 days later. Briefly, blood was collected in 3.5 mL EDTA plastic tubes (Becton Dickinson) via antecubital vein puncture using a 21-gauge needle, without a tourniquet. Within 15 min from blood collection, platelet-depleted plasma was prepared by double centrifugation using an Eppendorf Centrifuge 5702R, equipped with a swing-out rotor and a radius of 132 mm (Eppendorf, Hamburg, Germany). The centrifugation parameters were as follows: 2500×*g*, 15 min, 23 °C, acceleration speed one, and no brake.

The first centrifugation step was done using 3.5 mL whole blood collection tubes. The supernatant was collected at 10 mm above the buffy coat. The second centrifugation step was performed with 1 mL of plasma in 10 mL polypropylene centrifuge tubes. Supernatant (platelet-depleted plasma) was collected 5 mm above the bottom of the tube, transferred into 10 mL polypropylene centrifuge tubes, mixed by pipetting, transferred to 0.5 mL Eppendorf tubes (Greiner Bio-One, Kremsmünster, Austria), and stored at − 80°C until analysis. Prior to analysis, the samples were thawed for 1 min in a water bath at 37 °C.

### Flow cytometry

To assess EV concentrations, we used flow cytometry, the most reliable technique for estimating the surface antigen expression of EVs. The concentration of (1) total particles, (2) EVs, and (3) lipoproteins within well-defined size and fluorescence ranges was measured using flow cytometry (A60-Micro, Apogee Flow Systems, Hertfordshire, UK). To ensure reproducibility, we completed the MIFlowCYt-EV template and added the supporting information (Supplemental File 2). Samples were diluted twofold to 1500-fold in Dulbecco’s phosphate-buffered saline (DPBS) to achieve a count rate of less than 3000 events/s to prevent swarm detection^45^. The diluted samples were measured for 120 s at a flow rate of 3.01 µL per min. The trigger threshold was set at 24 arbitrary units of the side-scatter detector, which corresponded to a side-scattering cross section of 7 nm^2^.

Total particle concentrations were defined as all particles exceeding the trigger threshold, which include EVs > 160 nm in diameter (assuming a core refractive index of 1.38, a shell refractive index of 1.48, and a shell thickness of 6 nm), lipoproteins (assuming a refractive index of 1.475^46^ > 120 nm in diameter, and protein complexes per mL of plasma.

EV concentrations were defined as particles with a diameter > 200 nm a refractive index (RI) < 1.41^47^, as determined by the flow cytometry scatter ratio (Flow-SR)^46^, and positive at the fluorescence detector(s) corresponding to the used label(s) per mL of plasma.

Lipoproteins were defined as particles with a diameter > 200 nm with a refractive index > 1.5^47^, as determined by Flow-SR^46^ per mL of plasma.

The validation of the flow cytometry method can be found in methodological guidelines to study extracellular vesicles and was described elsewhere^44–47^.

### Statistical analysis

Statistical analyses were performed using the STATISTICA software version 13 (StatSoft, Kraków, Poland). The Shapiro–Wilk test was used to test the normality of the distribution of variables. The variables are expressed as medians with the 25th and 75th percentiles. Friedman's test (*p* = 0.002) with Dunn's test as a post hoc test was used to assess the changes in individual parameters because of apheresis sessions. Univariate correlations were assessed using standardized Spearman coefficients. *P* values below 0.05 were considered statistically significant.

## Results

### EV concentration

**Figure 1:**
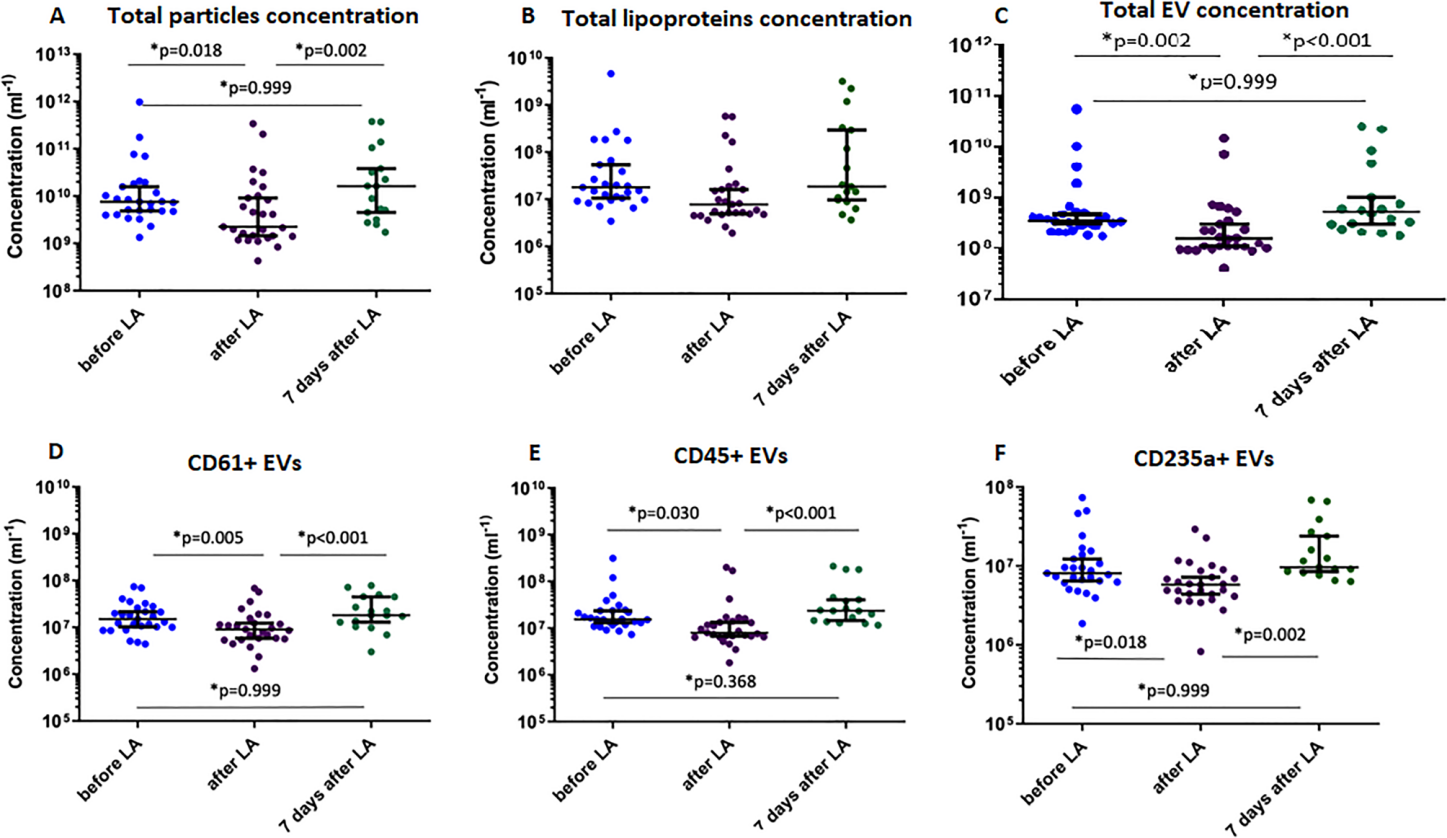
Impact of lipoprotein apheresis (LA) on the concentration of extracellular vesicles (EVs) measured with flow cytometry. Concentrations of extracellular vesicles (EVs) in plasma for (**A**) the total concentration of particles > 100 nm in diameter, (**B**) the total concentration of lipoprotein particles (diameter > 200 nm, refractive index between 1.5 and 1.6), (**C**) the total concentration of EVs (diameter > 200 nm, refractive index [RI] < 1.41), and the concentration of (**D**) CD61 + EVs, (**E**) CD45 + EVs, and (**F**) CD235a + EVs (diameter > 200 nm, refractive index < 1.41). The line and error bars indicate the median value and the 5 and 95 percentiles, respectively. All measured particle concentrations decreased significantly after LA procedure and rebounded to the basis levels after 7 days. Abbreviations: CD—cluster of differentiation*,* RI—refractive index.

The changes in total particle concentration (diameter >  ~ 100 nm), total lipoproteins concentration (diameter > 200 nm, 1.50 < RI < 1.60), total EV concentration (diameter > 200 nm, RI < 1.41), concentrations of EVs derived from erythrocytes (CD235a + ; diameter > 200 nm, RI < 1.41), leukocytes (CD45+; diameter > 200 nm, RI < 1.41) and platelets (CD61 + , PEVs; diameter > 200 nm, RI < 1.41) before, immediately after, and 7 days after LA are presented in Fig. 1 (panels A–F). All presented values significantly decreased immediately after LA and restored seven days after LA. Total particle concentration was reduced by 59.8% (IQR 37.8–77.3; *p* = 0.018) (Fig. 1A). LA resulted in a reduction of total lipoprotein concentration by 58.9% (IQR 9.8–79.8; *p* > 0.05) (Fig. 1B). Total EV concentration was lowered by 55,6% (47.6 – 67.8; *p* = 0.002) (Fig. 1C). The CD61 + EV concentration was reduced by 42.7% after LA (IQR 12.8–54.7; *p* = 0.005) (Fig. 1D). LA resulted in a 42.6% reduction of CD45 + EV concentration (IQR 29.7–54.1; *p* = 0.030) (Fig. 1E). The CD235a + concentration decreased by 26.7% (IQR 1.0–62.7; *p* = 0.018) (Fig. 1F).

### Biochemical parameters

**Figure 2:**
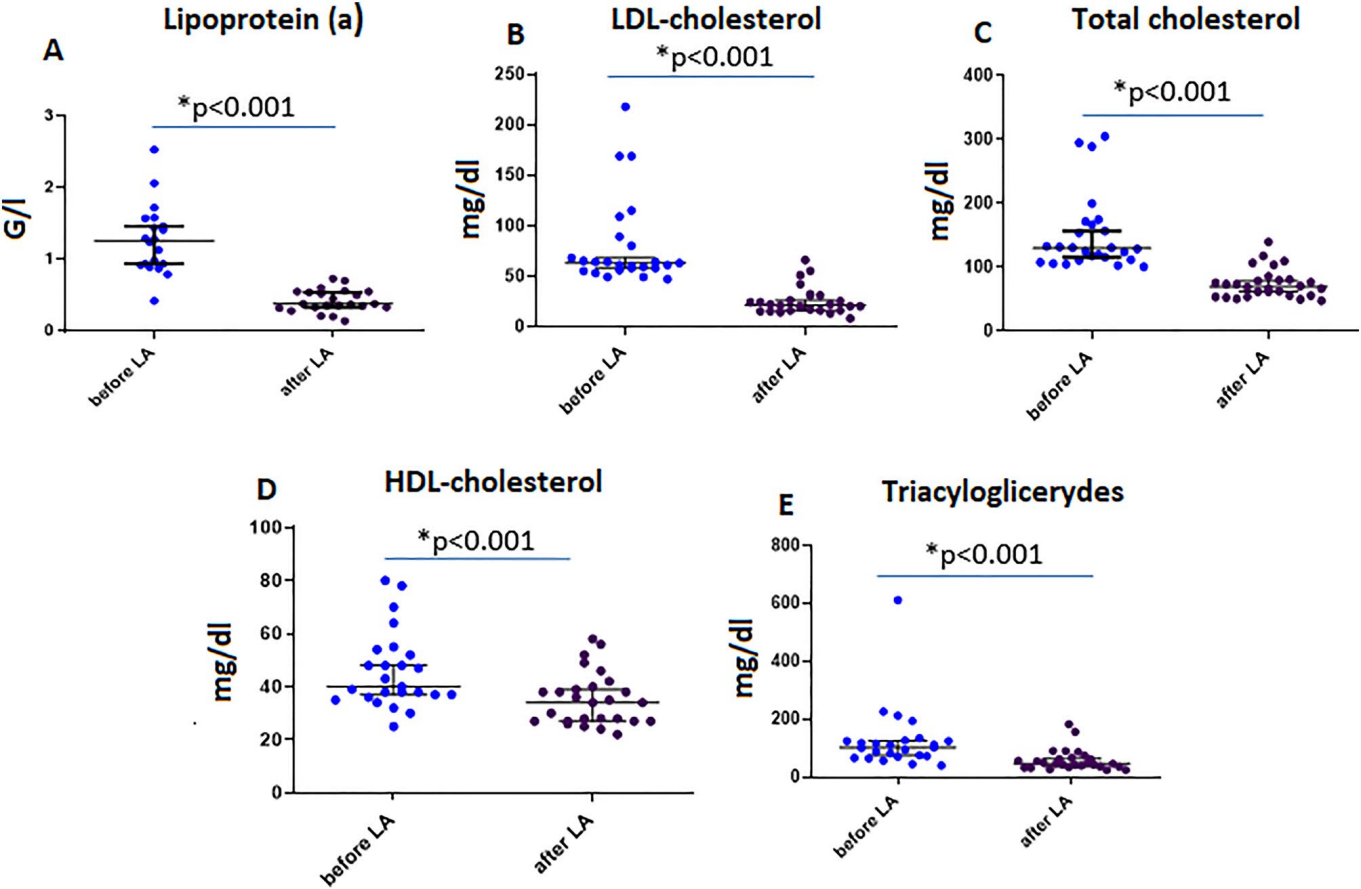
Impact of lipoprotein apheresis (LA) on lipid parameters. Concentrations of lipid parameters in plasma for (**A**) lipoprotein(a), (**B**) LDL-cholesterol, (**C**) Total cholesterol, (**D**) HDL-cholesterol, (**E**) triacyloglicerydes. The line and error bars indicate the median value and the 5 and 95 percentiles, respectively. All measured particle concentrations decreased significantly after LA procedure. Abbreviations: LDL-cholesterol—low-density lipoprotein cholesterol, HDL-cholesterol—high-density lipoprotein cholesterol.

The concentrations of Lp(a) and all lipid parameters significantly decreased immediately after LA and restored seven days after LA (Table 1). The impact of LA on the Lp(a) and lipid parameters is shown in Fig. 2 (Panels A-E). The apheresis procedure led to a 64.5% (IQR 58–71) reduction of Lp(a) concentration (Fig. 2A). LDL-C concentration was lowered by 67% (IQR 65- 74) (Fig. 2B). Substantial reductions were also noted in total cholesterol (Fig. 2C), HDL-C (Fig. 2D) and triglyceride concentrations (Fig. 2E) with percentage reductions as follow 51.7% (IQR 44.5–53), 22% (IQR 18.8–25.6), 49.5% (IQR 40.8–60), respectively.

**Table 1:** Biochemical parameters before and after lipoprotein apheresis (LA).

Parameter	Pre-apheresis, median value (interquartile range)	Post-apheresis, median value (interquartile range)	7 days after apheresis, median value (interquartile range)
Lp(a) [G/l]	1.26 (0.9–1.54)	0.38 (0.33–0.55)	1.34 (1.21–1.47
LDL-C [mg/dl]	63 (5 –84.5)	21 (16–31)	65 (51–88)
TC [mg/dl]	128 (110.5–161)	68 (55– 9)	128.5 (120–172)
HDL-C [mg/dl]	40 (36.5–53)	34 (27–40)	41.5 (38–49)
TG [mg/dl]	103 (72–126)	47 (36–67)	103 (89–175)

Lp(a)—lipoprotein (a), LDL-C—low density lipoprotein cholesterol, TC—total cholesterol, HDL-C—high density lipoprotein cholesterol, TG—triglycerides.

### Changes in EV concentrations and lipid parameters

Almost all percentage values of changes in EV concentration of various origin were significantly correlated with each other except 1.5 < RI < 1.6 lipoproteins with CD61+ and CD235a + EVs. Also, total EVs did not correlate with CD235a + EVs (Table 2). In relation to changes in lipid parameters, significant correlations were observed in the case of percentage changes in CD235a + EVs with total cholesterol (R = 0.476; *p* = 0.014), LDL-C (R = 0.449; *p* = 0.02), and triglycerides (R = 0.441; *p* = 0.02). Changes in CD45 + EVs correlated only with triglycerides (R = 0.440; *p* = 0.02). Total and CD61 + EVs percentage changes did not correlate with the lipid parameters.

**Table 2:** Univariate correlations of percentage changes in extracellular vesicle (EV) concentrations of various origins.

	% of changes				
	EVs (CD61+)	EVs (CD45+)	EVs (CD235a+)	1.5 < RI < 1.6 EVs	Total EVs
% of changes					
EVs (CD61 +)	–	R = 0.747; *p* < 0.001	R = 0.582; *p* = 0.001	Non-significant	R = 0.496; *p* = 0.007
EVs (CD45+)	R = 0.747; *p* < 0.001	–	R = 0.423; *p* = 0.02	R = 0.437 *p* = 0.02	R = 0.645 *p* < 0.001
EVs (CD235a+)	R = 0.582; *p* = 0.001	R = 0.423; *p* = 0.02	–	Non-significant	Non-significant
1.5 < RI < 1.6 EVs	Non-significant	R = 0.436 *p* = 0.02	Non-significant	–	R = 0.532 *P* = 0.003
Total EVs	R = 0.496; *p* = 0.007	R = 0.645 *p* < 0.001	Non-significant	R = 0.532 *P* = 0.003	–

Correlations were assessed using standardized Spearman coefficients.

LA—lipoprotein apheresis, CD—cluster of differentiation*,* EVs—extracellular vesicles, RI—refractive index.

## Discussion

The main findings of our study are that (1) LA immediately reduces the concentrations of all subcellular particles > 100 nm, including EVs, (2) this reduction is independent of the cellular origin of EVs, (3) EV concentrations are restored 7 days after LA.

We hypothesize that LA-induced removal of EVs might be one of the mechanisms contributing to the favorable effects of LA. The reduction of EV concentration has been previously considered as a pleiotropic LA effect^30^. Lowering EV concentration could be related to a decrease in CV risk, as numerous publications have underlined the negative role of EVs in the pathogenesis of ASCVD and their unfavorable content among ASCVD patients. It has been shown that EVs isolated from the blood differed between patients with ASCVD and healthy individuals. EVs isolated from CVD patients had the potential to induce endothelial dysfunction, whether those obtained from healthy patients did not^48,49^ .

In our study, CD61 + EVs (platelet EVs, PEVs) were reduced by 42.7% during a single LA procedure. PEVs exhibit procoagulant actions, which play a major role in platelet aggregation and thrombus formation. PEVs also promote the formation of atheromatous plaques and accumulate in the lipid core of the plaques^50^. Higher PEV concentrations are observed in patients with ASCVD. Interestingly, there was a lack of association between PEV concentration and CVD risk factors among asymptomatic patients with atherosclerosis, what could be explained by the fact that thrombogenesis is mildly expressed at early stages of ASCVD^51^.

EVs derived from leukocytes (CD45+) were another subtype of EVs analyzed in our study. We noted CD45 + EV concentrations reduction by 42.6% during LA. EVs originating from all types of inflammatory cells play a complex role in the immune system as they possibly trigger systemic inflammatory response^52^. Progressive inflammation-induced impairment of endothelial cell function promotes atherosclerotic degeneration and plays an important role in ASCVD development^53^. In addition, leukocyte-derived EVs were proven to be higher in patients with preclinical atherosclerosis and positively associated with CVD risk factors^51^. Hence, lowering the concentration of EVs, especially leukocytes, will contribute to beneficial effects by reducing local inflammation and subsequently reducing cardiovascular risk^53^.

The third type of EVs investigated in our study was erythrocyte-derived EVs (CD235a+) with a transmembrane glycoprotein expressed on erythrocytes. The role of CD235a + EVs in ASCVD pathogenesis remains unclear. Although higher concentrations of erythrocyte-derived EVs were observed among patients with myocardial infarction compared to that in the control group, its usefulness as a future marker is unfortunately small, and further studies are needed^54^. Undoubtedly, the findings of our study indicate a 26.7% reduction in CD235a + EVs during the LA procedure. We also observed a correlation between CD235a + EVs and the total cholesterol, HDL-C, and triglyceride concentrations. However, considering the low clinical usefulness of EVs derived from erythrocytes, this association seems to have no practical benefits.

Elevated Lp(a) concentration is more prevalent in patients with FH, compared to general population. Those two determinants additively increase a CV risk. Li et al. showed that subjects undergoing coronary angiography with confirmed or probable FH, assessed by DLCN score, had higher Lp(a) concentration compared to those without FH. Lp(a) + FH phenotype translated into earlier and more severe course of CVD^55^. The LA therapy is an effective therapeutic option also for patients with hyper-Lp(a) and coexisting FH. Among our patients with elevated Lp(a) treated by LA, only three had confirmed HeFH. However, we did not observe an earlier or more severe course of CVD in those subjects. It should be also highlighted, that our patients were treated with cascade filtration technique (MONET system). Although various LA techniques, including direct absorption (DALI), specific immunoadsorption (IMA), dextran sulphate adsorption (DSA), heparin-induced extracorporeal LDL precipitation (HELP) exert comparable effects on lipid parameters^56^, the impact on C-reactive protein, leukocyte count or coagulation parameters differed, depending on the LA system^57^. The changes in EV concentrations in patients treated with DALI (8 patients), whole blood dextrane sulfate adsorption (1 patient) and plasma dextrane sulfate adsorption (3 patients) were similar^42^. Further studies are needed to investigate the effect of various LA systems on EV concentrations.

EVs are involved in inflammation, thrombosis, and neo-angiogenesis and participate in all stages of atherosclerotic plaque formation^38^. Initially, an increase in EV concentration results from prolonged exposure to damaging factors, leading to endothelial cell activation and apoptosis^58^. Further accumulation of EVs increases plaque instability. In the case of plaque rupture, an increase in EV concentration, mainly in leukocytes, platelets, endothelial cells, and erythrocytes is observed^59^.

In another study LA was shown to affect platelet-derived and annexin V-positive EVs. Likewise, the authors hypothesized an additional apheresis effect, as it removes EVs involved in procoagulation pathways^42^. However, the use of annexin V to identify EVs populations has been questioned, while annexin V requires calcium ions to bind EVs surface and calcium ions are simultaneously involved in the coagulation pathway as a cofactor^60^. Since then, the technology for EV measurements has evolved. In our study, we employed pre-analytical and analytical techniques using flow cytometry to measure concentrations of EVs from platelets (CD61+), leukocytes (CD45+), and erythrocytes (CD235a +). Currently, this is the most reliable technique for estimating the surface antigen expression of EVs. To our knowledge, this is the first study to show the beneficial effects of LA on different subtypes of EVs among patients with hyper-Lp(a) and ASCVD. LA is currently an effective method for cardiovascular risk reduction among patients with genetically determined hyper-Lp(a) who, despite maximally tolerated lipid-lowering medications and low LDL-C concentrations, still have a high residual risk of CV events. In our study, we did not find any correlation between EVs and Lp(a) concentration changes. Therefore, we assume that reducing EV concentration is an independent, additional LA benefit and aim to state that this finding may be related to the reduction in the rate of cardiovascular events. Moreover, we have also shown that EV concentrations rebound within 7 days after apheresis, which may suggest the need to shorten the interval between LA procedures.

### Limitations

A limitation of our study is that we cannot confirm the direct association between the LA-induced removal of EVs and beneficial LA effects. Further research is needed to evaluate EV concentrations during 1 and 6 day after LA.

We would like to emphasize that the strength of our study is the flow cytometry method applied to measure concentrations of platelet-derived (CD61+), leukocyte-derived (CD45+), and erythrocyte-derived (CD235a+) EVs. Although we measured only a fraction of all EVs in plasma, owing to assay controls and calibration we know the lower diameter limit (> 200 nm) and fluorescence intensity ranges of the EVs that we measured. Consequently, our data can be reproduced by other hospitals in the near future.

## Conclusion

The findings of our study revealed that LA in patients with elevated Lp(a) and ASCVD led to (1) a substantial reduction in EV concentration, (2) regardless the cell origination. (3) This effect is transient, as we observed EVs concentrations rebound after 7 days of monitoring. The last conclusion indicates a need to shorten the intervals between LA procedures to less than one week. The removal of EVs with procoagulant and proinflammatory properties may be the additional, pleiotropic LA benefit. Future research is needed to indicate, whether this mechanism underlies the additional favorable effect of the LA procedure. Future studies should also address the pathophysiological insights and biomarkers for ASCVD mediated by high Lp(a) concentrations.

To summarize, our study is a reliable source of data, covers previously unexplored research field and provides basis for further research.

### Supplementary Information

Supplementary Information 1.

Supplementary Information 2.

## Data Availability

Data are to be shared upon request (Medical University of Gdansk, amickiewicz@gumed.edu.pl).

## Abbreviations

LA: Lipoprotein apheresis
ASCVD: Atherosclerotic cardiovascular disease
hyper-Lp(a): Hyperlipoproteinemia(a)
EVs: Extracellular vesicles
Lp(a): Lipoprotein (a)
MACE: Major adverse cardiovascular events
FH: Familial hypercholesterolemia

## References

[1] Saeedi R, Frohlich J (2016). Lipoprotein (a), an independent cardiovascular risk marker. Clin Diabetes Endocrinol.

[2] Tipping RW, Ford CE, Simpson LM, Walldius G, Jungner I, Folsom AR (2009). Lipoprotein(a) concentration and the risk of coronary heart disease, stroke, and nonvascular mortality. JAMA.

[3] Patel AP, Wang M, Pirruccello JP, Ellinor PT, Ng K, Kathiresan S (2021). Lp(a) (Lipoprotein[a]) concentrations and incident atherosclerotic cardiovascular disease: New insights from a large national biobank. Arterioscler Thromb Vasc Biol.

[4] Coassin S, Kronenberg F (2022). Lipoprotein(a) beyond the kringle IV repeat polymorphism: The complexity of genetic variation in the LPA gene. Atherosclerosis.

[5] Schmidt K, Noureen A, Kronenberg F, Utermann G (2016). Structure, function, and genetics of lipoprotein (a). J Lipid Res.

[6] Utermann G (1979). The mysteries of lipoprotein(a). Science.

[7] Banach M, Surma S, Toth PP (2023). The year in cardiovascular disease—the year of new and prospective lipid lowering therapies: Can we render dyslipidemia a rare disease by 2024?. Arch. Med. Sci..

[8] Farnier M, Chagué F, Maza M, Bichat F, Masson D, Cottin Y (2022). High lipoprotein(a) levels predict severity of coronary artery disease in patients hospitalized for acute myocardial infarction: Data from the French RICO survey. J Clin Lipidol.

[9] Panza GA, Blazek O, Tortora J, Saucier S, Fernandez AB (2023). Prevalence of lipoprotein(a) measurement in patients with or at risk of cardiovascular disease. J Clin Lipidol.

[10] Tsimikas S, Bhatia HS, Erlinge D (2023). Clinical trials to improve outcomes in patients with elevated Lp(a) undergoing PCI: The time has arrived. J Clin Lipidol.

[11] Simantiris S, Antonopoulos AS, Papastamos C, Benetos G, Koumallos N, Tsioufis K (2023). Lipoprotein(a) and inflammation- pathophysiological links and clinical implications for cardiovascular disease. J Clin Lipidol.

[12] Kronenberg F, Mora S, Stroes ESG, Ference BA, Arsenault BJ, Berglund L (2022). Lipoprotein(a) in atherosclerotic cardiovascular disease and aortic stenosis: A European Atherosclerosis Society consensus statement. Eur Heart J.

[13] Waldmann E, Parhofer KG (2016). Lipoprotein apheresis to treat elevated lipoprotein (a). J Lipid Res.

[14] Nugent AK, Gray JV, Gorby LK, Moriarty PM (2020). Lipoprotein apheresis: First FDA indicated treatment for elevated lipoprotein(a). J. Clin. Cardiol..

[15] Szymański FM, Mickiewicz A, Dzida G, Gorczyca-Głowacka I, Kozłowski D, Widecka K (2022). Management of dyslipidemia in Poland: Interdisciplinary expert position statement endorsed by the polish cardiac society working group on cardiovascular pharmacotherapy: The fourth declaration of sopot. Cardiol J.

[16] Banach M, Burchardt P, Chlebus K, Dobrowolski P, Dudek D, Dyrbuś K (2021). PoLA/CFPiP/PCS/PSLD/PSD/PSH guidelines on diagnosis and therapy of lipid disorders in Poland 2021. Arch. Med. Sci..

[17] Mickiewicz A, Marlega J, Kuchta A, Bachorski W, Cwiklinska A, Raczak G (2021). Cardiovascular events in patients with familial hypercholesterolemia and hyperlipoproteinaemia (a): Indications for lipoprotein apheresis in Poland. J Clin Apher.

[18] Schettler VJJ, Peter C, Zimmermann T, Julius U, Roeseler E, Schlieper G (2021). The German lipoprotein apheresis registry (GLAR)—more than 7 years on. Atherosclerosis.

[19] Thompson GR (2008). Recommendations for the use of LDL apheresis. Atherosclerosis.

[20] Connelly-Smith, L., Alquist, C. R., Aqui, N. A., Hofmann, J. C., Klingel, R., Onwuemene, O. A., *et al*. Guidelines on the use of therapeutic apheresis in clinical practice - evidence-based approach from the writing committee of the American Society for Apheresis: The Ninth Special Issue. *J. Clin. Apher.* **38**, 277–278 (2023). 10.1002/JCA.22043. 10.1002/jca.22043 37017433

[21] Franchini M, Capuzzo E, Liumbruno GM (2016). Lipoprotein apheresis for the treatment of elevated circulating levels of lipoprotein(a): a critical literature review. Blood Transfusion.

[22] Jaeger, B. R., Richter, Y., Nagel, D., Heigl, F., Vogt, A., Roeseler, E., *et al*. Longitudinal cohort study on the effectiveness of lipid apheresis treatment to reduce high lipoprotein(a) levels and prevent major adverse coronary events. 10.1038/ncpcardio1456 (2009). 10.1038/ncpcardio1456 19234501

[23] Marlȩga-Linert J, Wartecka-Zielińska K, Wydra D, Fijałkowski M, Gruchała M, Mickiewicz A (2023). Case report: Lipoprotein apheresis reduces the risk of cardiovascular events and prolongs pregnancy in a woman with severely elevated lipoprotein(a), cardiovascular disease, and a high risk of preeclampsia. Front Med (Lausanne).

[24] Mickiewicz A, Marlęga-Linert J, Czapiewska M, Marcinkowska M, Krzesińska A, Kuchta A (2023). Fatty acid analysis in serum of patients with elevated lipoprotein(a) and cardiovascular disease undergoing lipoprotein apheresis. J Clin Lipidol.

[25] Stefanutti C, Pisciotta L, Favari E, Di Giacomo S, Vacondio F, Zenti MG (2020). Lipoprotein(a) concentration, genetic variants, apo(a) isoform size, and cellular cholesterol efflux in patients with elevated Lp(a) and coronary heart disease submitted or not to lipoprotein apheresis: An Italian case-control multicenter study on Lp(a). J Clin Lipidol.

[26] Rubba P, Iannuzzi A, Postiglione A, Scarpato N, Montefusco S, Gnasso A (1990). Hemodynamic changes in the peripheral circulation after repeat low density lipoprotein apheresis in familial hypercholesterolemia. Circulation.

[27] Pleiotropic effects of regular lipoprotein-apheresis. *Atheroscler. Suppl.* **30**, 122–127. 10.1016/J.ATHEROSCLEROSISSUP.2017.05.032 (2017). 10.1016/j.atherosclerosissup.2017.05.032 29096827

[28] Mickiewicz, A., Kreft, E., Kuchta, A., Wieczorek, E., Marlȩga, J., Ćwiklińska, A., *et al*. The impact of lipoprotein apheresis on oxidative stress biomarkers and high-density lipoprotein subfractions. *Oxid. Med. Cell. Longev*. (2020). 10.1155/2020/9709542. 10.1155/2020/9709542 PMC7428943 32832012

[29] Sinzinger H, Steiner S, Derfler K (2017). Pleiotropic effects of regular lipoprotein-apheresis. Atheroscler Suppl.

[30] Schettler VJJ, Schettler E (2022). Beyond cholesterol—pleiotropic effects of lipoprotein apheresis. Therap. Apheresis Dial..

[31] Sedgwick AE, D’Souza-Schorey C (2018). The biology of extracellular microvesicles. Traffic.

[32] Zhang X, Wu Y, Cheng Q, Bai L, Huang S, Gao J (2022). Extracellular vesicles in cardiovascular diseases: Diagnosis and therapy. Front Cell Dev Biol.

[33] Microparticles and coronary artery disease - Nomura - 1997 - American Journal of Hematology - Wiley Online Library n.d. https://onlinelibrary.wiley.com/doi/abs/10.1002/%28SICI%291096-8652%28199712%2956%3A4%3C296%3A%3AAID-AJH19%3E3.0.CO%3B2-7?sid=nlm%3Apubmed. Accessed 7 March 2023.

[34] Martin-Ventura JL, Roncal C, Orbe J, Blanco-Colio LM (2022). Role of extracellular vesicles as potential diagnostic and/or therapeutic biomarkers in chronic cardiovascular diseases. Front Cell Dev Biol.

[35] Chong, S. Y., Lee, C. K., Huang, C., Ou, Y. H., Charles, C. J., Richards, A. M., *et al*. Extracellular vesicles in cardiovascular diseases: Alternative biomarker sources, therapeutic agents, and drug delivery carriers. *Int. J. Mol. Sci.* 20. 10.3390/IJMS20133272 (2019). 10.3390/ijms20133272 PMC6650854 31277271

[36] Han C, Yang J, Sun J, Qin G (2022). Extracellular vesicles in cardiovascular disease: Biological functions and therapeutic implications. Pharmacol Ther.

[37] Gąsecka A, Van Der Pol E, Nieuwland R, Stępień E (2018). Extracellular vesicles in post-infarct ventricular remodelling. Kardiol Pol.

[38] Badimon L, Padro T, Arderiu G, Vilahur G, Borrell-Pages M, Suades R (2022). Extracellular vesicles in atherothrombosis: From biomarkers and precision medicine to therapeutic targets. Immunol Rev.

[39] Katopodis J, Kolodny L, Jy W, Horstman L, de Marchena E, Tao JG (1997). Platelet microparticles and calcium homeostasis in acute coronary ischemias. Am J Hematol.

[40] Gąsecka A, Rogula S, Eyileten C, Postuła M, Jaguszewski MJ, Kochman J (2020). Role of P2Y receptors in platelet extracellular vesicle release. Int J Mol Sci.

[41] Suades R, Padró T, Alonso R, López-Miranda J, Mata P, Badimon L (2014). Circulating CD45+/CD3+ lymphocyte-derived microparticles map lipid-rich atherosclerotic plaques in familial hypercholesterolaemia patients. Thromb Haemost.

[42] Connolly KD, Willis GR, Datta DBN, Ellins EA, Ladell K, Price DA (2014). Lipoprotein-apheresis reduces circulating microparticles in individuals with familial hypercholesterolemia. J Lipid Res.

[43] Krzych ŁJ, Czok M, Putowski Z (2020). Is antimicrobial treatment effective during therapeutic plasma exchange? Investigating the role of possible interactions. Pharmaceutics.

[44] Coumans FAW, Brisson AR, Buzas EI, Dignat-George F, Drees EEE, El-Andaloussi S (2017). Methodological guidelines to study extracellular vesicles. Circ Res.

[45] Buntsma NC, Shahsavari M, Gąsecka A, Nieuwland R, van Leeuwen TG, van der Pol E (2023). Preventing swarm detection in extracellular vesicle flow cytometry: A clinically applicable procedure. Res Pract Thromb Haemost.

[46] van der Pol E, de Rond L, Coumans FAW, Gool EL, Böing AN, Sturk A (2018). Absolute sizing and label-free identification of extracellular vesicles by flow cytometry. Nanomedicine.

[47] de Rond, L., Libregts, S. F. W. M., Rikkert, L. G., Hau, C. M., van der Pol, E., Nieuwland, R., *et al*. Refractive index to evaluate staining specificity of extracellular vesicles by flow cytometry. *J. Extracell. Vesicles* **5**, 80. 10.1080/20013078.2019.1643671 (2019). 10.1080/20013078.2019.1643671 PMC6713200 31489142

[48] Boulanger CM, Scoazec A, Ebrahimian T, Henry P, Mathieu E, Tedgui A (2001). Circulating microparticles from patients with myocardial infarction cause endothelial dysfunction. Circulation.

[49] Jansen F, Li Q, Pfeifer A, Werner N (2017). Endothelial- and Immune cell-derived extracellular vesicles in the regulation of cardiovascular health and disease. Basic Translat. Sci..

[50] Shantsila E, Kamphuisen PW, Lip GYH (2010). Circulating microparticles in cardiovascular disease: Implications for atherogenesis and atherothrombosis. J. Thromb. Haemostasis.

[51] Chironi G, Simon A, Hugel B, Del PM, Gariepy J, Freyssinet JM (2006). Circulating leukocyte-derived microparticles predict subclinical atherosclerosis burden in asymptomatic subjects. Arterioscler Thromb Vasc Biol.

[52] Buzas EI (2022). The roles of extracellular vesicles in the immune system. Nat. Rev. Immunol..

[53] Akhmerov A, Parimon T (2022). Extracellular vesicles, inflammation, and cardiovascular disease. Cells.

[54] Yuan Y, Maitusong M, Muyesai N (2020). Association of endothelial and red blood cell microparticles with acute myocardial infarction in Chinese: A retrospective study. Ann Palliat Med..

[55] Li S, Wu NQ, Zhu CG, Zhang Y, Guo YL, Gao Y (2017). Significance of lipoprotein(a) levels in familial hypercholesterolemia and coronary artery disease. Atherosclerosis.

[56] Lipoprotein Apheresis - PubMed n.d. https://pubmed.ncbi.nlm.nih.gov/28402616/. Accessed 20 Dec 2023.

[57] Julius U, Frind A, Tselmin S, Kopprasch S, Poberschin I, Siegert G (2008). Comparison of different LDL apheresis methods. Expert Rev Cardiovasc Ther.

[58] Jansen F, Nickenig G, Werner N (2017). Extracellular vesicles in cardiovascular disease. Circ Res.

[59] Boulanger CM, Loyer X, Rautou PE, Amabile N (2017). Extracellular vesicles in coronary artery disease. Nat Rev Cardiol.

[60] Kuiper M, van de Nes A, Nieuwland R, Varga Z, van der Pol E. Reliable measurements of extracellular vesicles by clinical flow cytometry. *Am. J. Reprod. Immunol.* **85**. 10.1111/AJI.13350 (2021). 10.1111/aji.13350 PMC7900981 32966654

