# Supplementary material for: Lipoprotein apheresis affects the concentration of extracellular vesicles in patients with elevated lipoprotein (a)
Source: Sci Rep. 2024 Feb 2;14:2762. doi: 10.1038/s41598-024-51782-5 (PMC10837138; doi:10.1038/s41598-024-51782-5)
Supplement: Supplementary file 1 — Supplementary Information 1. [file 41598_2024_51782_MOESM1_ESM.docx]

Supplemental Table 1. Clinical and biochemical characteristics of the study group

| Patient's number | 301 | 302 | 304 | 305 | 306 | 307 | 308 | 310 | 311 | 312 | 313 | 314 | 315 | 316 | 317 | 318 | 319 | 320 | 321 | 322 | 323 | 324 |
| --- | --- | --- | --- | --- | --- | --- | --- | --- | --- | --- | --- | --- | --- | --- | --- | --- | --- | --- | --- | --- | --- | --- |
| Gender | F | F | F | M | M | F | M | M | F | M | M | F | M | M | M | M | F | M | M | F | F | F |
| Lp(a) before first LA, mg/dl | 137 | 113 | 199 | 129 | 100 | 245 | 119 | 182 | 100 | 143 | 221 | 152 | 133 | 111 | 121 | 329 | 124 | 136 | 118 | 145 | 146 | 129 |
| Age of first LA | 55 | 66 | 46 | 55 | 69 | 37 | 66 | 69 | 52 | 51 | 63 | 39 | 58 | 62 | 49 | 44 | 46 | 61 | 72 | 66 | 63 | 64 |
| Year of starting LA | 2013 | 2021 | 2016 | 2020 | 2021 | 2020 | 2020 | 2021 | 2020 | 2020 | 2017 | 2021 | 2021 | 2021 | 2021 | 2021 | 2021 | 2021 | 2021 | 2021 | 2021 | 2022 |
| CAD | 1 | 1 | 1 | 1 | 1 | 1 | 1 | 1 | 1 | 1 | 1 | 1 | 1 | 1 | 1 | 1 | 1 | 1 | 1 | 1 | 1 | 1 |
| CAD, age | 48 | 45 | 46 | 54 | 40 | 37 | 56 | 54 | 37 | 51 | 61 | 39 | 51 | 62 | 46 | 35 | 46 | 51 | 50 | 59 | 63 | 63 |
| ACS | 0 | 1 | 1 | 1 | 1 | 1 | 0 | 1 | 1 | 0 | 0 | 1 | 0 | 0 | 1 | 1 | 1 | 1 | 1 | 0 | 0 | 1 |
| ACS, age of first | NA | 45 | 46 | 54 | 40 | 37 | NA | 54 | 37 | NA | NA | 39 | NA | NA | 46 | 35 | 46 | 51 | 50 | NA | NA | 63 |
| PCI | 3 | 3 | 0 | 2 | 2 | 3 | 6 | 3 | 1 | 2 | 3 | 2 | 2 | 2 | 6 | 9 | 3 | 4 | 5 | 3 | 2 | 1 |
| PCI, age of first | 48 | 52 | NA | 54 | 47 | 37 | 56 | 54 | 44 | 51 | 61 | 39 | 56 | 63 | 46 | 35 | 46 | 51 | 57 | 59 | 63 | 63 |
| CABG | 0 | 1 | 0 | 0 | 2 | 0 | 0 | 0 | 0 | 0 | 0 | 0 | 1 | 0 | 0 | 0 | 0 | 0 | 1 | 0 | 0 | 0 |
| CABG, age | NA | 46 | ND | NA | 40 | NA | NA | NA | NA | NA | ND | NA | 51 | NA | NA | NA | NA | NA | 72 | NA | NA | NA |
| TIA | 0 | 0 | 0 | 0 | 0 | 0 | 0 | 0 | 0 | 0 | 0 | 0 | 0 | 0 | 0 | 0 | 0 | 0 | 0 | 0 | 0 | 0 |
| Stroke | 0 | 1 | 0 | 0 | 0 | 0 | 0 | 0 | 0 | 0 | 0 | 0 | 0 | 0 | 0 | 0 | 0 | 0 | 0 | 0 | 0 | 0 |
| Stroke, age of first | NA | 57 | NA | NA | NA | NA | NA | NA | NA | NA | NA | NA | NA | NA | NA | NA | NA | NA | NA | NA | NA | NA |
| Carotid artery disease | 0 | 1 | 0 | 0 | 0 | 0 | 0 | 0 | 0 | 0 | 0 | 0 | 0 | 1 | 0 | 0 | 0 | 0 | 0 | 0 | 0 | 0 |
| Peripheral artery disease | 0 | 1 | 0 | 0 | 0 | 0 | 0 | 0 | 1 | 0 | 0 | 0 | 0 | 1 | 0 | 0 | 0 | 0 | 0 | 0 | 0 | 0 |
| Revascularization of carotid or peripheral artery | 0 | 1 | 0 | 0 | 0 | 0 | 0 | 0 | 1 | 0 | 0 | 0 | 0 | 1 | 0 | 0 | 0 | 0 | 0 | 0 | 0 | 0 |
| BMI | 24 | 22 | 30 | 27 | 27 | 26 | 22 | 32 | 24 | 29 | 29 | 33 | 29 | 26 | 26 | 28 | 34 | 32 | 30 | 29 | 28 | 25 |
| HeFH | 1 | 1 | 0 | 0 | 0 | 0 | 0 | 0 | 0 | 0 | 1 | 0 | 0 | 0 | 0 | 0 | 0 | 0 | 0 | 0 | 0 | 0 |
| Diabetes | NA | 0 | 0 | 0 | 1 | 0 | 0 | 1 | 0 | 0 | 0 | 0 | 0 | 0 | 0 | 0 | 1 | 1 | 0 | 0 | 0 | 0 |
| Hypertension | 1 | 1 | 0 | 0 |  | 0 | 1 | 1 | 0 | 1 | 1 | 0 | 1 | 1 | 0 | 0 | 1 | 1 | 1 | 1 | 0 | 1 |
| Smoking history | 1 | 1 | 0 | 1 | 1 | 0 | 0 | 1 | 1 | 0 | 0 | 0 | 1 | 1 | 1 | 0 | 1 | 1 | 0 | 1 | 1 | 0 |
| Family history of early ASCVD in 1st-degree relative | 1 | 0 | NA | 1 | 1 | 1 | 1 | 1 | 1 | 1 | 1 | 1 | 1 | 1 | 1 | 1 | 1 | 1 | 1 | 0 | 1 | 1 |
| CKD | 0 | 0 | 0 | 0 | 0 | 0 | 0 | 1 | 0 | 0 | 0 | 0 | 0 | 0 | 0 | 0 | 0 | 0 | 0 | 0 | 0 | 0 |
| LVEF (%) | 60 | 65 | 50 | 55 | 53 | 60 | 55 | 50 | 65 | 60 | 65 | ND | 60 | 60 | 55 | ND | 55 | 55 | 50 | 60 | ND | 60 |

Abbreviations: ACS- acute coronary syndrome, CAD- coronary artery disease, CABG- coronary artery bypass graft, CKD- chronic kidney disease, F- female, HeFH- heterozygous FH, M- male, LA - lipoprotein apheresis, : Lp(a) – lipoprotein (a), LDL-C – low density lipoprotein cholesterol, LVEF- left ventricle ejection fraction, TC – total cholesterol, HDL-C – high density lipoprotein cholesterol, PAD- Peripheral artery disease, PCI – percutaneus intervention, TG – triglycerides, TIA- transient ischemic attack
