# Supplementary material for: Lipoprotein apheresis affects the concentration of extracellular vesicles in patients with elevated lipoprotein (a)
Source: Sci Rep. 2024 Feb 2;14:2762. doi: 10.1038/s41598-024-51782-5 (PMC10837138; doi:10.1038/s41598-024-51782-5)
Supplement: Supplementary file 2 — Supplementary Information 2. [file 41598_2024_51782_MOESM2_ESM.docx]

**Assessment of lipoprotein apheresis-induced extracellular vesicles plasma concentrations changes in patients with elevated Lp(a)**

**Lipoprotein apheresis affects the concentration of extracellular vesicles in patients with elevated Lp(a)**

**Supplementary File 2. MIFlowCyt checklist**

| **Requirement** | **Please Include Requested Information** |
| --- | --- |
| 1.1. Purpose | The purpose of this experiment was to determinate the plasma concentration of different types of extracellular vesicles (EVs) in patients undergoing lipoprotein apheresis (LA) due to elevated lipoprotein (a) and cardiovascular disease and to investigate LA impact on EVs.  Flow cytometry was used to measure platelet (CD61+)-derived EVs, leukocyte (CD45+)-derived EVs and erythrocyte (CD235a+)-derived EVs concentration at 3 time points – immediately before LA, immediately after LA and 7 days after LA. We hypothesized that lipoprotein apheresis would lower EV concentration, which could bring additional clinical LA benefit. |
| 1.2. Keywords | Extracellular vesicles, lipoprotein apheresis, lipoprotein (a) |
| 1.3. Experiment variables | EDTA blood samples were centrifugated twice, according to blood processing protocol to obtain platelet-depleted plasma. The obtained plasma was used as input for flow cytometry (A60-Micro, Apogee Flow Systems, Hertfordshire, UK) to identify the EVs. |
| 1.4. Organization name and address | Lipoprotein Apheresis Unit  First Department of Cardiology  Medical University of Gdansk  M. Skłodowskiej-Curie 3a  80-210 Gdansk  Poland  Amsterdam University Medical Centers  Location University of Amsterdam  Meibergdreef 9  1105 AZ Amsterdam  The Netherlands |
| 1.5. Primary contact name and email address | Agnieszka Mickiewicz; amickiewicz@gumed.edu.pl |
| 1.6. Date or time period of experiment | April 2021 – May 2022 |
| 1.7. Conclusions | LA resulted in an immediate substantial reduction in platelet (CD61+), leukocyte (CD45+), and erythrocyte (CD235a+) EVs concentrations. There was no correlation between the LA-induced reduction in EVs and Lp(a) concentrations. All EVs subtypes returned to the baseline concentrations after 7 days. |

| 1.8. Quality control measures | All samples were measured using flow cytometry (A60-Micro, Apogee Flow Systems, Hertfordshire, UK). Samples were diluted 2-fold to 1500-fold in Dulbecco’s phosphate-buffered saline (DPBS) to achieve a count rate of less than 3000 events/s to prevent swarm detection. (1) The diluted samples were measured for 120 s at a flow rate of 3.01 µL per min. The trigger threshold was set at 24 arbitrary units of the side-scatter detector, which corresponded to a side-scattering cross section of 7 nm^2^. |
| --- | --- |
| 1.9 Other relevant experiment information | The experiment was performed among 22 patients undergoing biweekly LA for hyper-Lp(a) and atherosclerotic cardiovascular disease (ASCVD). The cascade filtration technique MONET was used in all patients. Different EVs subtypes were measured using flow cytometry before LA, directly after LA and 7 days later. |
| 2.1.1.1. Sample description | Frozen double centrifugated plasma (section 2.1.1.2) from 22 patients undergoing biweekly LA for hyper-Lp(a) and ASCVD. This plasma was assessed in flow cytometry (A60-Micro, Apogee Flow Systems, Hertfordshire, UK) to identificate the EVs. |
| 2.1.1.2. Biological sample source description | Peripheral venous blood samples were collected from fasting patients according to recent guidelines to study EVs. (2) Each patient had blood collected at three points in time: immediately before and after a single LA procedure, and then 7 days later. Briefly, blood was collected in 3.5 mL EDTA plastic tubes (Becton Dickinson) via antecubital vein puncture using a 21-gaugae needle, without a torniquet. After a maximum of 15 minutes of blood collection, platelet-depleted plasma was prepared by double centrifugation using an Eppendorf Centrifuge 5702R, equipped with a swing-out rotor and a radius of 132 mm (Eppendorf, Hamburg, Germany). The centrifugation parameters were as follows: 2500 × g, 15 min, 23 °C, acceleration speed one, and no brake.  The first centrifugation step was done using 3.5 mL whole blood collection tubes. The supernatant was collected at 10 mm above the buffy coat. The second centrifugation step was performed with 1 mL of plasma in 10 mL polypropylene centrifuge tubes. Supernatant (platelet-depleted plasma) was collected 5 mm above the buffy coat, transferred into 10 mL polypropylene centrifuge tubes, mixed by pipetting, transferred to 0.5 mL Eppendorf tubes (Greiner Bio-One, Kremsmünster, Austria), and stored at −80°C until analysis. Prior to analysis, the samples were thawed for 1 min in a water bath at 37 °C.  The concentration of (1) total particles, (2) EVs, and (3) lipoproteins within well-defined size and fluorescence ranges was measured using flow cytometry (A60-Micro, Apogee Flow Systems, Hertfordshire, UK). Samples were diluted 2-fold to 1500-fold in Dulbecco’s phosphate-buffered saline (DPBS) to achieve a count rate of less than 3000 events/s to prevent swarm detection. (1) The diluted samples were measured for 120 s at a flow rate of 3.01 µL per min. The trigger threshold was set at 24 arbitrary units of the side-scatter detector, which corresponded to a side-scattering cross section of 7 nm^2^.  Total particle concentrations were defined as all particles exceeding the trigger threshold, which include EVs >160 nm in diameter (assuming a core refractive index of 1.38, a shell refractive index of 1.48, and a shell thickness of 6 nm), lipoproteins (assuming a refractive index of 1.475 (3) >120 nm in diameter, and protein complexes per mL of plasma.  EV concentrations were defined as particles with a diameter >200 nm a refractive index (RI) <1.41 (4), as determined by the flow cytometry scatter ratio (Flow-SR) (3), and positive at the fluorescence detector(s) corresponding to the used label(s) per mL of plasma.  Lipoproteins were defined as particles with a diameter >200 nm with a refractive index >1.5 (4), as determined by Flow-SR (3) per mL of plasma. |

| 2.1.1.3. Biological sample source organism description | 22 patients undergoing biweekly LA for hyper-Lp(a) and ASCVD. |
| --- | --- |
| 2.2 Sample characteristics | Plasma is expected to contain different subtypes of EVs. Single lipoprotein apheresis procedure is expected to reduce the EV concentrations. Flow cytometry was used to measure platelet (CD61+)-derived EVs, leukocyte (CD45+)-derived EVs and erythrocyte (CD235a+)-derived EV concentration. |
| 2.3. Sample treatment description | Samples were prepared according to protocol to obtain platelet-depleted plasma. They were stored at −80°C until analysis. Prior to analysis, the samples were thawed for 1 min in a water bath at 37 °C. |
| 2.4. Fluorescence reagent(s) description | **Table S1.1** contains an overview of the staining reagents.    Anti-CD41-PE (Biocytex, Marseille, France), CD61-APC  (Invitrogen, Waltham, MA), CD62p-PE (Beckman Coulter, Brea,  CA) and anti-CD235a-FITC (Dako, Amstelveen, The  Netherlands) were pre-diluted in DPBS (Corning, Amsterdam, The Netherlands) and centrifuged at 18.890 x g to remove antibody aggregates.    Twenty µl of each sample was added to 30 µl HEPES buffer (137 mmol/L NaCl (6404, Merck Millipore), 20 mmol/L Hepes (10110,  Merck Millipore), 5.6 mmol/L D-glucose (8337, Merck |

|  | Millipore), 0.1%, BSA (A9647, 0.1%, Sigma-Aldrich, St. Louis, MO, 3.3 mmol/L fc NaH_2_PO_4_.H_2_O (6345, Merck Millipore), 2.7 mmol/L fc, KCl (24936, Merck Millipore), and 1 mmol/L fc MgCl_2_.6H_2_O (5833, Merck Millipore) in Milli-Q (Baxter, TKF7114, Deerfield, IL)).  Subsequently, 5 µl pre-diluted anti-CD235a-FITC, 10 µl prediluted mix of anti-CD61-APC and anti-CD41-PE or 10 µl prediluted mix of anti-CD61-APC and anti-CD62p-PE was added. After a 30 minute incubation in the dark at room temperature (RT), samples were fixated with 200 µl HEPES buffer/0.3% paraformaldehyde (PFA; 104005, Merck Millipore) for 1 hour in the dark at RT. In case samples exceeded an event rate of > 10,000 evts/sec during measurements, samples were further diluted in HEPES buffer/0.3% PFA. |
| --- | --- |
| 3.1. Instrument manufacturer | Apogee Flow Systems, |
| 3.2. Instrument model | A60-Micro |
| 3.3. Instrument configuration and settings | The concentration of (1) total particles, (2) EVs, and (3) lipoproteins within well-defined size and fluorescence ranges was measured using flow cytometry (A60-Micro, Apogee Flow Systems, Hertfordshire, UK). Samples were diluted 2-fold to 1500-fold in Dulbecco’s phosphate-buffered saline (DPBS) to achieve a count rate of less than 3000 events/s to prevent swarm detection. (1) The diluted samples were measured for 120 s at a flow rate of 3.01 µL per min. The trigger threshold was set at 24 arbitrary units of the side-scatter detector, which corresponded to a side-scattering cross section of 7 nm^2^.  Total particle concentrations were defined as all particles exceeding the trigger threshold, which include EVs >160 nm in diameter (assuming a core refractive index of 1.38, a shell refractive index of 1.48, and a shell thickness of 6 nm), lipoproteins (assuming a refractive index of 1.475 (3) >120 nm in diameter, and protein complexes per mL of plasma.  EV concentrations were defined as particles with a diameter >200 nm a refractive index (RI) <1.41 (4), as determined by the flow cytometry scatter ratio (Flow-SR) (3), and positive at the fluorescence detector(s) corresponding to the used label(s) per mL of plasma.  Lipoproteins were defined as particles with a diameter >200 nm with a refractive index >1.5 (4), as determined by Flow-SR (3) per mL of plasma. |
| 4.1. List-mode data files | A summary of all flow cytometry scatter plots and gates applied are available via:  <https://doi.org/10.6084/m9.figshare.c.6126783.v1> |
| 4.2. Compensation description | No compensation was required, because no fluorophore combinations were used that have overlapping emission spectra. |
| 4.3. Data transformation details | Light scattering calibration and fluorescence calibration were applied, as indicated below. Concentrations reported in the manuscript describe the number of particles that fulfil the gating criteria per mL.  **Light scatter calibration**  We used Rosetta Calibration (v1.28, Exometry BV, Amsterdam, The Netherlands) to relate side scattering (SSC) to the effective scattering cross section and diameter of platelets and ery-ghosts.    **Figure S1.1** shows a print screen of the scatter calibration settings. Platelets were modelled as core-shell particles with a core refractive index of 1.38, a shell refractive index of 1.48, and |
|  | a shell thickness of 6 nm. Ery-ghosts were modelled as core-shell particles with a core refractive index of 1.35, a shell refractive index of 1.48, and a shell thickness of 6 nm. For each measurement, we added the SSC cross sections and particle diameters to the flow cytometry datafiles. The SSC trigger threshold corresponds to a side scattering cross section of 27 nm^2^.    **Fluorescence calibration**  Calibration of the fluorescence detectors from arbitrary units (a.u.) to molecules of equivalent soluble fluorochrome (MESF) was accomplished using 2-µm APC Quantitation Beads (2321-175, BD), Quantum^TM^ FITC-5 MESF Beads (13734, Bangs  Laboratories, Inc Fishers, IN) and SPHERO^TM^ PE Calibration  Particle Kit, 3.0-3.4 µm (ECFP-F2-5K, AK01, Spherotech Inc., Irma Lee Circle, IL). Calibrations of the APC, FITC and PE detectors were performed on 2022-01-24. For each measurement, we added fluorescent intensities in MESF to the flow cytometry data files by custom-build software (MATLAB R2018a) using the following equation:   \| I(MESF) =10𝑎∙log10 I(a.u.)+𝑏 \| Equation S1 \| \| --- \| --- \|   where I, is the fluorescence intensity, and a and b are the slope and the intercept of the linear fits respectively, see **Table S1.2**. |
| 4.4.1. Gate description | All gates were manually set using FlowJo (v 10.8.1, Flowjo, Ashland, OR). Gates for platelets, activated platelets and eryghosts were set based on fluorescence intensities (CD61+/CD41+, CD61+/CD62p+ and CD235a+, respectively) and light scattering intensities. An example of the gating strategy is depicted in **Figure S1.2.1, S1.2.2. and S1.2.3.** |
| 4.4.2. Gate statistics | The concentration of positive events was calculated by taking into account the flow rate, measurement time and dilutions performed during sample preparation. |
| 4.4.3. Gate boundaries | An overview of all gates can be found in the data summary file via: <https://doi.org/10.6084/m9.figshare.c.6126783.v1> |

Lipoprotein apheresis (LA); atherosclerotic cardiovascular disease (ASCVD); hyperlipoproteinemia(a) (hyper-Lp(a)); extracellular vesicles (EVs); Lipoprotein (a) (Lp(a)); CD: cluster of differentiation; EDTA: Ethylenediamine tetraacetic acid; RI: refractive index

**References**

1. Van Der Pol E, Van Gemert MJC, Sturk A, Nieuwland R, Van Leeuwen TG. Single vs. swarm detection of microparticles and exosomes by flow cytometry. J Thromb Haemost [Internet]. 2012 May [cited 2023 Mar 17];10(5):919–30. Available from: https://pubmed.ncbi.nlm.nih.gov/22394434/

2. Coumans FAW, Brisson AR, Buzas EI, Dignat-George F, Drees EEE, El-Andaloussi S, et al. Methodological Guidelines to Study Extracellular Vesicles. Circ Res [Internet]. 2017 May 12 [cited 2023 Mar 7];120(10):1632–48. Available from: https://pubmed.ncbi.nlm.nih.gov/28495994/

3. van der Pol E, de Rond L, Coumans FAW, Gool EL, Böing AN, Sturk A, et al. Absolute sizing and label-free identification of extracellular vesicles by flow cytometry. Nanomedicine. 2018 Apr 1;14(3):801–10.

4. de Rond L, Libregts SFWM, Rikkert LG, Hau CM, van der Pol E, Nieuwland R, et al. Refractive index to evaluate staining specificity of extracellular vesicles by flow cytometry. J Extracell Vesicles [Internet]. 2019 Dec 1 [cited 2023 Mar 7];8(1). Available from: /pmc/articles/PMC6713200/

**Table S1.1: Overview of staining reagents.**  Characteristics being measured, analyte, analyte detector, reporter, isotype, clone, concentration, manufacturer, catalog number and lot number of used staining reagents.

| **Characteristic measured** | **Analyte** | **Analyte detector** | **Reporter** | **Isotype** | **Clone** | **Concentration during staining (µg mL^-1^)** | **Manufacturer** | **Catalog number** | **Lot number** |
| --- | --- | --- | --- | --- | --- | --- | --- | --- | --- |
| Integrin | Human CD61 | Anti-human CD61 antibody | APC | IgG1 | VI-PL2 | 0.69 | Invitrogen | 17-0619-42 | 2284236 |
| Integrin | Human CD41 | Anti-human CD41 antibody | PE | IgG1 | PL2-49 | 0.63 | Biocytex | 5112-  PE100T | 091251 |
| Glycoprotein | Human CD235a | Anti-human CD235a antibody | FITC | IgG1 | JC159 | 4.55 | Dako | F0870 | 20064863 |
| Selectin | Human CD62p | Anti-human CD62p antibody | PE | IgG1 | CLB-  Thromb/6 | 1.04 | Beckman  Coulter | IM1759U | 200053 |

APC: Allophycocyanin; FITC: Fluorescein Isothiocyanate; IgG: Immunoglobulin G; PE: Phycoerythrin

**Table S1.2: Overview of fluorescence calibrations**

|  | Calibration date | Slope | Intercept | R^2^ |
| --- | --- | --- | --- | --- |
| APC | 2022-01-24 | 1.3014 | -1.539 | 0.9902 |
| PE | 2022-01-24 | 0.9966 | 0.4940 | 1 |
| FITC | 2022-01-24 | 1.0737 | 1.2195 | 0.9994 |

**Figure S1.1.**


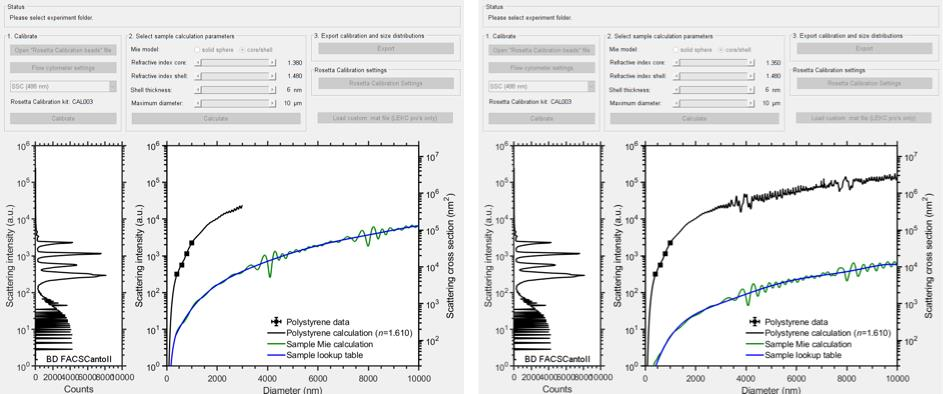


**Figure S1.1.** Side scatter calibration of the FACSCanto II. To relate scatter to the approximate diameter of (activated) platelets (left), we assumed platelets to be core-shell particles with a core refractive index of 1.38, a shell refractive index of 1.48, and a shell thickness of 6 nm. To relate scatter to the approximate diameter of ery-ghosts (right), we assumed ery-ghosts to be core-shell particles with a core refractive index of 1.35, a shell refractive index of 1.48, and a shell thickness of 6 nm.

**Figure S1.2.1.**


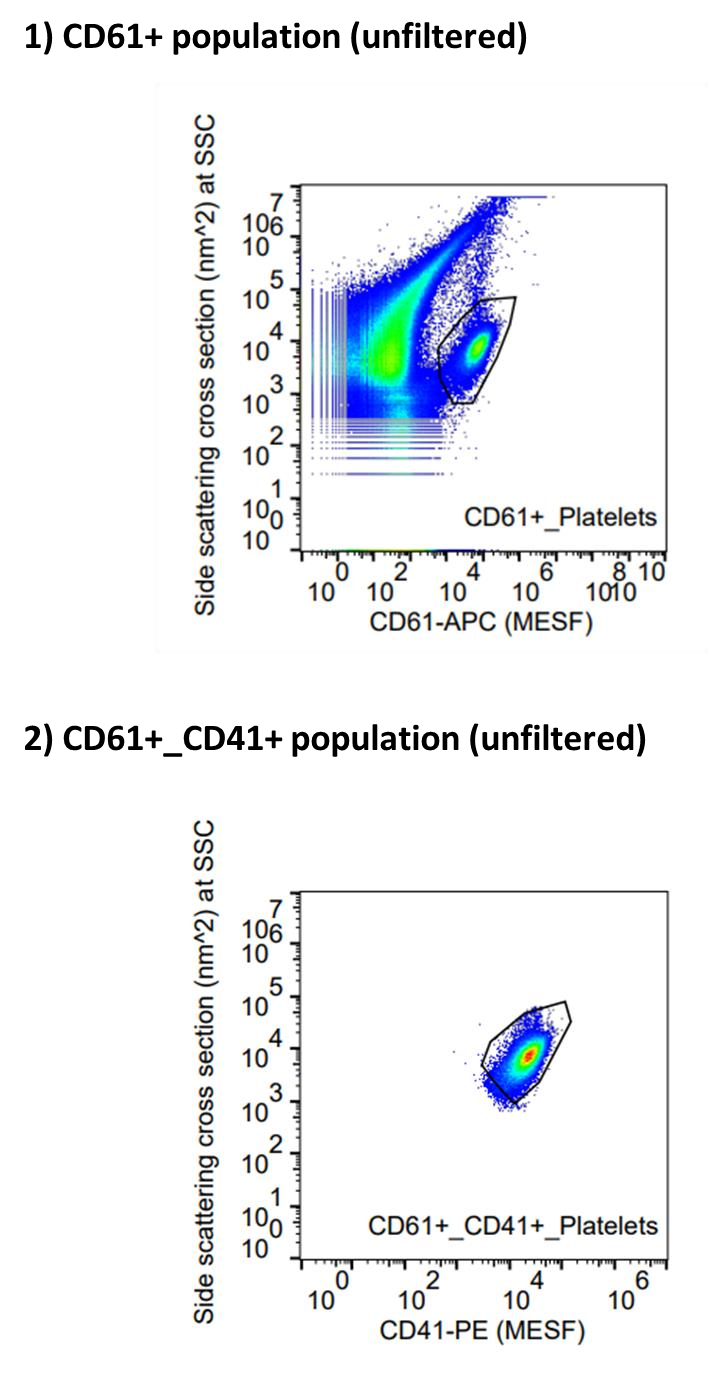


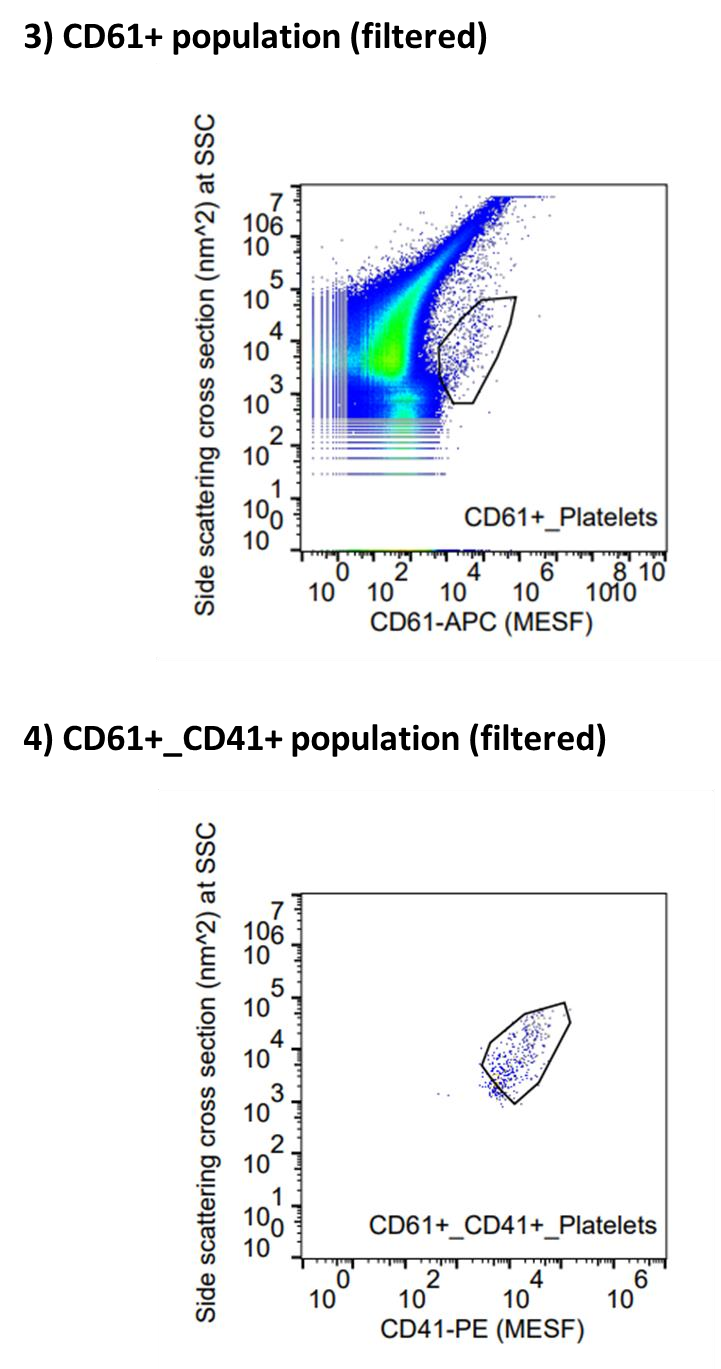


**Figure S1.2.1.** Gating strategy of platelets measured by the FACSCanto II. The plots show side scatter cross sections vs. fluorescence. (Panel 1, 2) Platelet gate on the unfiltered plasma sample. The CD61-APC+/CD41-PE+ population is defined as platelets. (Panel 3, 4) Platelet gate on the filtered plasma sample. The CD61-APC+/CD41-PE+ population is defined as platelets.

**Figure S1.2.2.**


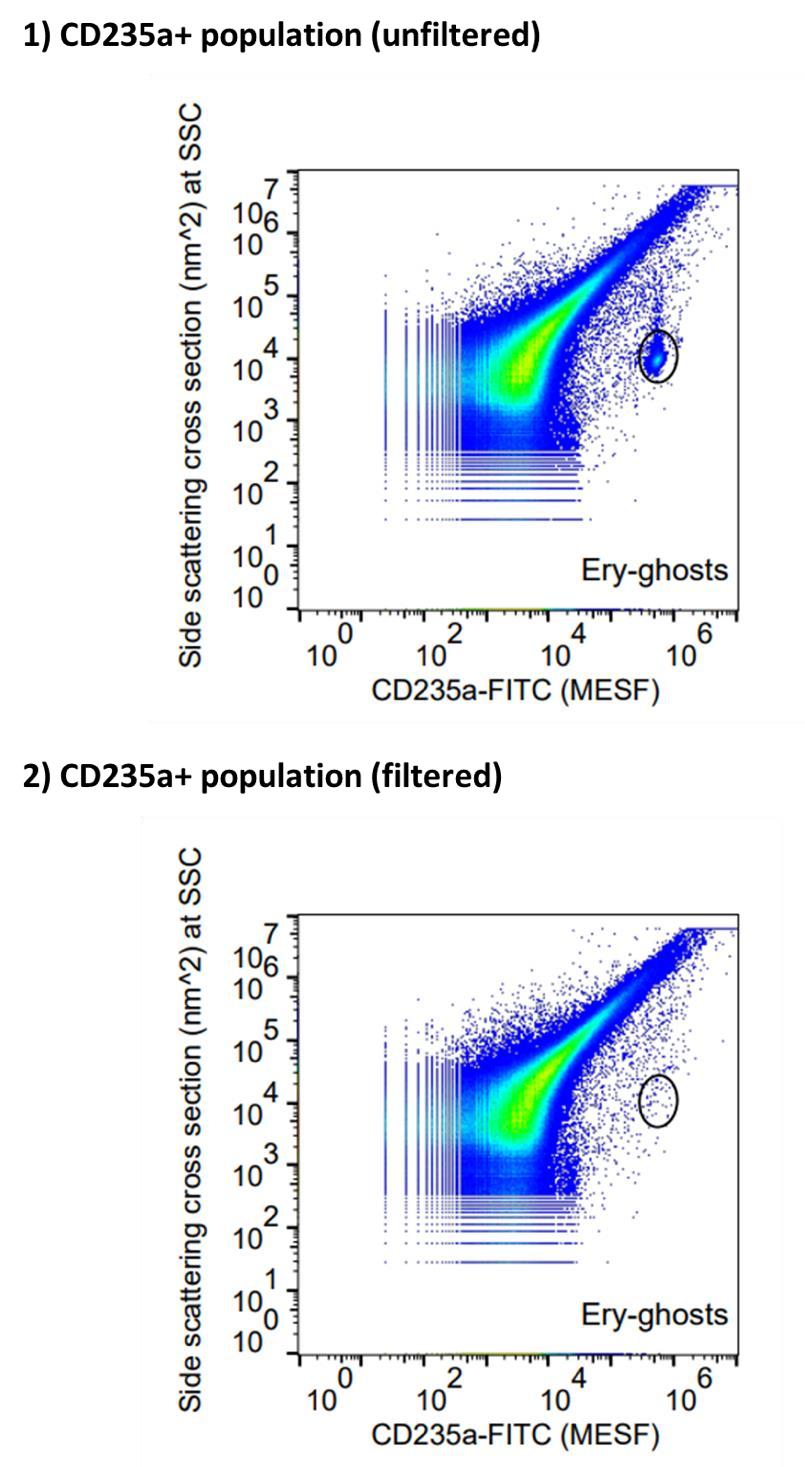


**Figure S1.2.2.** Gating strategy for erythrocyte ghosts (ery-ghosts) measured by the

FACSCanto II. The plots show side scatter cross sections vs. fluorescence for unfiltered (panel 1) and filtered (panel 2) plasma samples. The CD235a-FITC+ population is defined as ery-ghosts.

**Figure S1.2.3.**


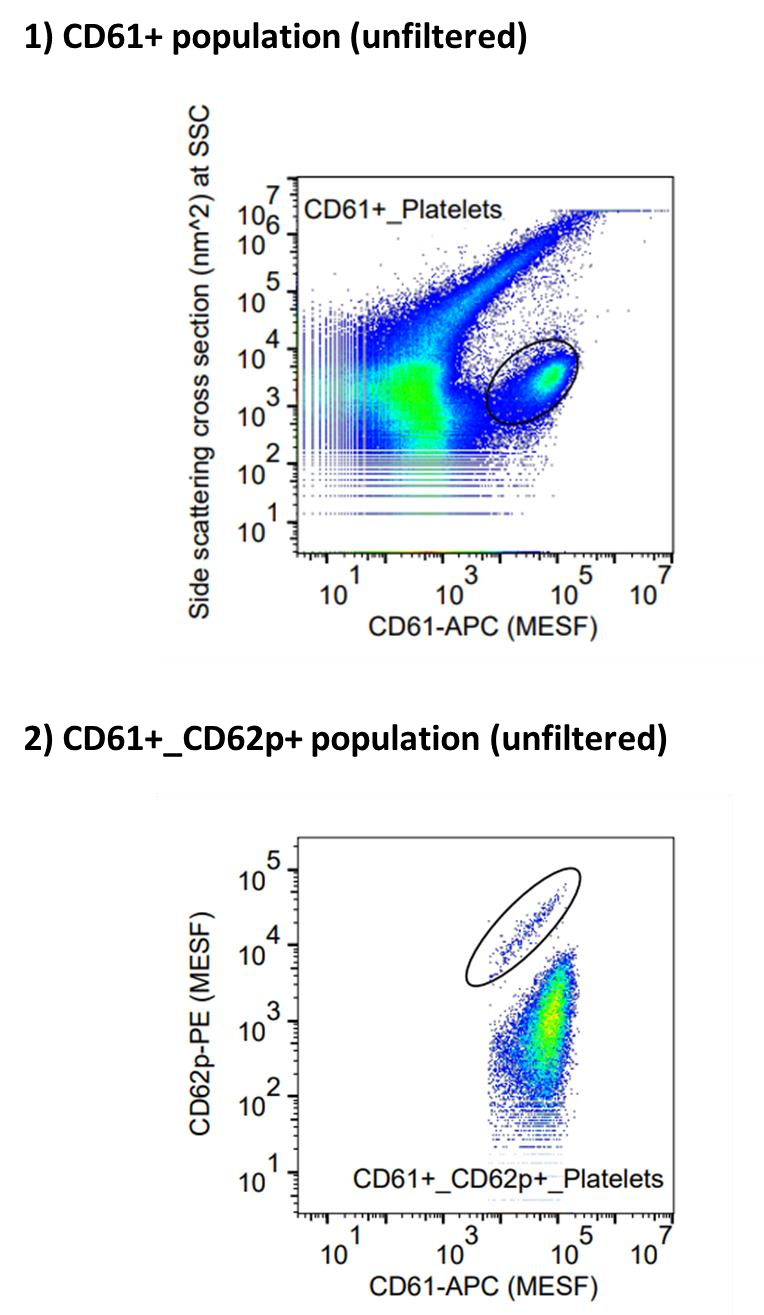


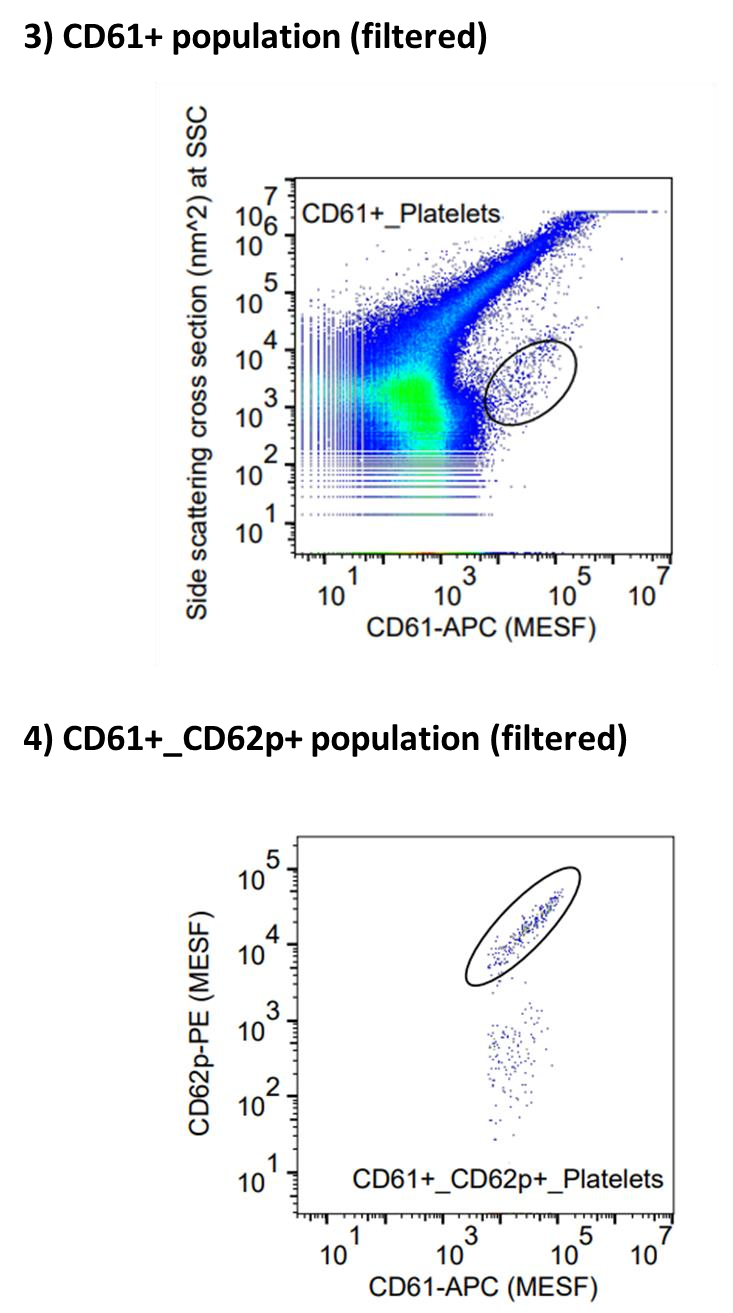


**Figure S1.2.3.** Gating strategy of activated platelets measured by the FACSCanto II. Panel 1 and 3 show plots of side scatter cross sections vs. fluorescence in filtered and unfiltered plasma samples, respectively. The CD61-APC+ population is defined as platelets. Panel 2 and 4 show fluorescence (CD61-APC, MESF) vs. fluorescence (CD62p-PE, MESF) in filtered and unfiltered samples, respectively. The CD61-APC+/CD62p-PE+ population is defined as activated platelets.
